# Metabolomic Analysis of the Effects of Adipose-Derived Mesenchymal Stem Cell Treatment on Rats With Sepsis-Induced Acute Lung Injury

**DOI:** 10.3389/fphar.2020.00902

**Published:** 2020-06-17

**Authors:** Yuqing Cui, Shaohua Liu, Xiaojuan Zhang, Xianfei Ding, Xiaoguang Duan, Zijia Zhu, Ji Zhang, Huoyan Liang, Dong Wang, Guojun Zhang, Zujiang Yu, Jianjun Yang, Tongwen Sun

**Affiliations:** ^1^General ICU, The First Affiliated Hospital of Zhengzhou University, Henan Key Laboratory of Critical Care Medicine, Zhengzhou Key Laboratory of Sepsis, Zhengzhou, China; ^2^Department of Pharmacy, The First Affiliated Hospital of Zhengzhou University, Zhengzhou, China; ^3^Department of Respiratory, The First Affiliated Hospital of Zhengzhou University, Zhengzhou, China; ^4^Department of Infectious Diseases, The First Affiliated Hospital of Zhengzhou University, Zhengzhou, China; ^5^Department of Anesthesiology, The First Affiliated Hospital of Zhengzhou University, Zhengzhou, China

**Keywords:** adipose-derived mesenchymal stem cells, metabolomics, acute lung injury, sepsis, inflammation

## Abstract

Given the high mortality associated with sepsis, there is an urgent need for a full understanding of sepsis pathophysiology and finding new therapeutic regimens. Adipose-derived mesenchymal stem cells (ADMSCs) has been proven to have anti-inflammatory effects and could be used to treat cecal ligation and puncture (CLP) induced lung and liver injury in septic rat models. In this study, we used metabolomics to investigate small molecule metabolites between CLP and ADMSCs treatment groups. Sixty SD rats were randomly assigned to the sham operation group (SC group), the CLP group, and the CLP+ADMSCs group (CLP-ADMSCs group). We used liquid mass spectrometry-chromatography to detect metabolic changes in plasma and lung tissues. Compared with the SC group, the metabolic profile of plasma and lung tissues changed significantly 24 h after CLP. Moreover, 22 and 11 main differential metabolites involved in amino acid and glycerophospholipid metabolism were found in plasma and lung tissues, respectively. After the rats were injected with ADMSCs, these differential metabolites were reverse-regulated both in plasma and lung tissues. Besides, ADMSCs improved the survival rate and down-regulated the concentration of TNF-α and IL-6 at 24 h after CLP. The correlational analysis between plasma of IL-6/TNF-α and metabolites suggested that acetylcholine, spermine, phenylalanine, threonine of plasma and phosphatidylcholine (36:4) of lung tissues were significantly associated with IL-6/TNF-α in CLP and CLP-ADMSCs groups. ADMSCs might reverse abnormal metabolic pathways by reducing anti-inflammatory factors in sepsis-induced ALI. Our findings may provide novel metabolic mechanism of ADMSCs therapy for sepsis.

## Introduction

Sepsis has been defined as a life-threatening organ dysfunction attributed to the host's dysregulated response to infection (Singer et al., 2016). Many pro-inflammatory cytokines, such as TNF-α and IL-6, reportedly triggered in sepsis, which caused an overwhelming immune response against the infection (Cecconi et al., 2018; Nowill et al., 2019). The treatment strategy for sepsis is a comprehensive treatment that mainly entails anti-microbial treatment, fluid resuscitation, application of glucocorticoids and vasoactive drugs, and mechanical ventilation (Rhodes et al., 2017). However, the mortality of acute lung injury (ALI) caused by sepsis continues to be high (Keeley et al., 2017). Therefore, it was very important to achieve a rapid diagnosis and therapeutic regimen for sepsis.

Fortunately, adipose-derived mesenchymal stem cells (ADMSCs) treatment has shown a lot of beneficial effects on sepsis. ADMSCs promote macrophage-mediated phagocytosis and secrete antibacterial peptides (Mei et al., 2010). Some studies reported mesenchymal stem cells (MSCs) could alleviate immune response and reduce mortality by down-regulating pro-inflammatory and up-regulating anti-inflammatory cytokines in sepsis (Condor et al., 2016; Ou et al., 2016). Besides, our previous research has suggested that ADMSCs secrete a large amount of sTNFR1 to block the activity of TNF-α, thereby reducing the expression of NF-κB, AP-1, and P38 MAPK in sepsis induced ALI (Ding et al., 2019). In addition, transforming growth factor-β (TGF-β) secreted by MSCs might reduce inflammatory response and improve phagocytosis (Liu et al., 2019). However, the mechanism of ADMSCs treatment still needs lot of experiments to explore.

As a branch of systematic biology, metabolomics analyzes changes in low-molecular-weight metabolites, including substances that may be continuously generated during disease development. Metabolomics data can accurately reflect the state of biological systems and promote our understanding of sepsis pathophysiology. A previous study (Su et al., 2015) have indicated that sepsis is comorbid with metabolic disorders, including abnormalities in amino acids, fatty acids, and bile acids. However, there has been no study on the metabolic effect of ADMSCs on sepsis. Thus, for the first time, we report metabolic changes caused by ADMSCs treatment of sepsis, and explore potential metabolic mechanism of ADMSCs treatment.

## Materials and Methods

### Instruments and Reagents

Exion LC AD series liquid chromatograph (SCIEX, USA), an X500R QTOF-MS mass spectrometer (SCIEX, Framingham, MA USA), and the high-speed refrigerated centrifuge 5810R (Eppendorf, Germany). Chromatography grade acetonitrile (Thermo Fisher Scientific, Waltham, MA USA), methanol (Sigma-Aldrich, St. Louis, MO USA), and pure water (Wahaha Company, China) were used. Rat TNF-α and IL-6 ELISA kits were acquired from CUSABIO (Wuhan, China). The Sprague-Dawley rat-derived ADMSCs originating from the inguinal fat were supplied by Cyagen Biotechnology (Yangzhou, China). Flow cytometry was used to assess ADMSCs that were positive for CD44, CD90, and CD29, but negative for CD34, CD11b, and CD45. ADMSCs were grown to the fourth generation based on the supplier's instructions.

### Animals, Grouping, and Experimental Procedure

Adult male Sprague-Dawley (SD) rats (180–250 g, 6–8 weeks old) were bought from Charles River (Beijing, China). All the experimental rats had access to food and water *ad libitum* and were housed in a room with a 12 h/12 h of day/night cycle for one week before the experiment. The temperature of the room was maintained at 20°C–25°C, and the relative humidity at 40% to 60%. Sixty SD rats were randomly assigned to the sham operation group (SC group), the cecal ligation, and the puncture model group (CLP group), and the CLP+ADMSCs group (CLP-ADMSCs group). One million ADMSCs were injected through the tail vein 1 h after the surgery in the CLP-ADMSCs group. The same volume of saline was injected in SC and CLP groups. The experiments were conducted in accordance with the guidelines developed by the National Institutes of Health (http://grants1.nih.gov/grants/olaw/) and approved by the Animal Care and Use Committee of the Zhengzhou University.

The CLP rat model was conducted as previously reported (Rittirsch et al., 2009). Briefly, 10% chloral hydrate was used to anesthetize the rats (350 mg/kg of body weight, intraperitoneally). The hair of the abdomen was shaved and the skin was disinfected with iodophor, prior to the operation, which was conducted under a sterile sheet. An abdominal midline incision of 1–2 cm was made, and the cecum was tightly ligated. The position of ligation was below the ileocecal valve to ensure intestine continuity. The middle and lower third of the cecum were ligated uniformly. Subsequently, 18-gauge needles were used to puncture two holes in the middle of ligated cecum. The cecum was squeezed gently and feces were exteriorized, and the cecum was returned to the abdomen. Sterile 5-0 and 3-0 needles were used to suture the muscle and skin layer, respectively. The SC group underwent the same procedure, but without ligation or puncture. For resuscitation after operation, saline (3mL/100 g) pre-heated to 37°C was injected subcutaneously. At the same time, a lamp was used to warm the rats for 30 min. For CLP-ADMSCs group, ADMSCs (1 × 10^6^) were injected through the tail vein 1 h after the operation. Then, the rats were placed in cages with access to food and water. Six rats of each group were anesthetized again 24 h after the surgery. Blood was collected from the abdominal aorta and centrifuged (2,500 rpm, 15 min) after 30 min standing at room temperature, to collect serum, which was then stored in liquid nitrogen. The chest was opened to expose the heart and inferior vena cava was cut. A perfusion needle was inserted into the apex of heart. Cold saline (100 mL–200 mL) was pushed rapidly into the left ventricle and aorta. The lungs became white, and one of the two lungs were extracted and placed in 4% paraformaldehyde overnight for histological analysis. The other lung was snap-frozen in liquid nitrogen. The lung tissues were embedded in paraffin and cut into 3- to 5-mm sections using a microtome. Sections were stained with hematoxylin and eosin and observed under a microscope (magnification: 100×) (Olympus, Japan).

### Plasma and Lung Samples Pretreatment

Plasma and lung samples were thawed on ice at 4°C. The plasma (50 μl) was transferred to an Eppendorf (EP) tube and 150 μl methanol and 10 μl the internal standard (0.5 μM/L cholic acid (CA-d4) and 0.5 μM/L chenodeoxycholic acid (CDCA-d4)) were added. The mixture was vortexed for 30 s and centrifuged for 10 min at 14,000 rpm. The supernatant was collected and injected into an auto-sampler container. Then, 20 mg lung samples and 200 μl pure water were added to an EP tube and vortexed for 2 min. 800 μl of pre-cooled methanol/acetonitrile (1:1) was added to the EP tube, which was then vortexed for 30 s and centrifuged (14,000 rpm, 10 min). The supernatant (900 μl) was collected and evaporated for 2.5 h, and 120 μl of 50% acetonitrile was added to dissolve it. The solution was vortexed for 30 s, centrifuged twice (14,000 rpm, 10 min) and vortexed three times (1500 rpm, 30 s). Finally, 100 μl supernatant was collected for injection. The quality control (QC) sample was mixed with 20 μl of each sample, and 200 μl of this mixture was used for QC analysis.

### Chromatography Conditions

The chromatographic column (ACQUITY UPLC HSS T3 C18, 2.1 mm × 100 mm, 1.7 μm; Waters, Milford, MA) was maintained at 30℃. The positive ion consisted of 0.1% formic acid in water (A phase) and acetonitrile (B phase), and the negative ion consisted of 5mM ammonium acetate in water (A phase) and 5 mM acetonitrile (B phase). A 14-min gradient elution method was used with a flow rate of 0.4 mL/min. The elution linear gradient started at 5% B phase, increased linearly to 30% in 1 min, 60% in the next 2 min, then increased linearly to 95% in 7 min, and remained constant for 2 min. Then, the B phase was recovered to 5% in 0.1 min and kept for 1.9 min. The injection volume was 3.0 μl.

### Mass Spectrometry

Data collection and processing were performed using the SCIEX OS 1.2 software (AB, Milford, MA). The ion source of the positive and negative ion scanning modes was electrospray ionization. The temperature and spray voltage of the ion source were set at 600°C, 5500 V (positive ion mode), and 600°C, -4500 V (negative ion mode), respectively. Declustering potential, auxiliary gas 1, auxiliary gas 2, and curtain gas were set at 80 V, 60, 60, and 35 psi, respectively. Collision energy was 20, 40, and 40 eV. The quality scanning range was *m/z* 50–1000 Da. Further, dynamic background subtraction and information-dependent acquisition were used for triggering low-level components to gather MS/MS.

### Multivariate Data Analysis and Identification of Potential Biomarkers

Data extraction, retention time correction, peak recognition, peak extraction, peak integration, peak alignment, and other pre-processing procedures were conducted using the Software MarkerView TM (version 1.4.1, Waters Co., Milford, MA, USA). The data matrix composed of retention time (RT), mass spectral (m/z), and peak intensity was produced after total normalization pretreatment. The Principal component analysis (PCA) was applied to draw score plots in order to evaluate the distribution of samples. The Orthogonal partial least squares discriminant analysis (OPLS-DA) was used to reduce dimension in the multi-dimensional complex data. The Variable importance projection (VIP) was computed to find differential metabolites between two groups. One-way ANOVA and the Tukey's *post hoc* test were used to analyze the data (SPSS software version 21.0, Chicago, IL). The metabolites satisfying VIP > 1.0, |FC| > 1.5, and *P* < 0.05 were considered as differential markers. Biomarkers identification and pathway enrichment analysis were investigated by combining human metabolism database (HMDB), the Kyoto Encyclopedia of genes and genomes (KEGG), MetaboAnalyst 4.0, other databases, and literature reports. Heat maps were generated with these significant metabolites by the R software (R version 3.5.3, heatmap package) to show the changes in trend. The links between plasma of IL-6/TNF-α and metabolites of plasma and lung tissues in CLP and CLP-ADMSCs groups were assessed using the R software (https://www.r-project.org/). The correlational analysis was conducted to obtain the correlation coefficient and *P* value between IL-6/TNF-α and metabolites. The correlation coefficient more than 0.75 and *P* < 0.2 were selected as differential metabolites significantly associated with IL-6/TNF-α.

## Results

### Pathology and Inflammatory Factor Levels in Septic ALI Model

**Figure 1:**
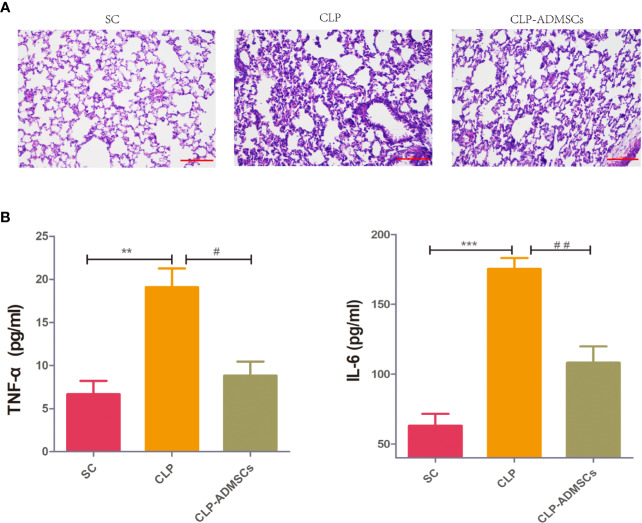
**(A)** Histology of the lungs of the SC, CLP, and ADMSCs groups after CLP 24h (n = 6). Hematoxylin and eosinstaining showed significantly enhanced inflammatory infiltration, edema, and bleeding in the CLP group. ADMSCs treatment improved these abnormalities (scale bar = 100 µm). H & E: Hematoxylin and eosin. **(B)** Effects of ADMSCs treatment on the systemic inflammatory response in CLP rats (n = 6). ELISA was used to detect plasma IL-6 and TNF-α concentration in each group at 24 h. *Compared with the SC group, ^#^compared with the CLP group; *, ^#^*P* < 0.05; **, ^##^*P* < 0.01; ***, ^###^*P* < 0.001.

The mortality of the SC, CLP, and CLP-ADMSCs groups was 0% (0/20), 40.0% (8/20), and 25.0% (5/20) at 24 h after operation. The mortality rate in the CLP group was higher than SC group (*P* = 0.003) and CLP-ADMSCs group (*P* = 0.501). The cecum of CLP group presented adhesion and necrosis, but these manifestations were markedly attenuated in the CLP-ADMSCs group. Hematoxylin and eosin staining of the lung tissues samples was used to evaluate lung injury (**Figure 1A**). Compared with SC group, the alveolar walls of the CLP group were found to be significantly thickened, and some of the alveolar structures were destroyed. The alveolar space was reduced and infiltrated with a lot of inflammatory cells in the CLP group, but these features were ameliorated in the CLP-ADMSCs group. Our previous study had successfully proved transplanted ADMSCs engrafted into the lung at 6, 24, and 72 h after a CLP procedure (Ding et al., 2019). Moreover, we detected plasma concentration of TNF‐α and IL-6 at 24 h after CLP and found levels of inflammatory factors up-regulated in the CLP group and significantly down-regulated in the CLP-ADMSCs group (**Figure 1B**).

### Plasma and Lung Chromatography Analysis

The plasma and lung metabolites of the three groups were analyzed, and the total ion chromatograms of plasma and the lung tissues samples were shown in **Supplementary Material** (**Supplementary Figures 1** and **2**). Compared with the CLP group, the peak pattern of the CLP-ADMSCs group was similar to SC group. The results suggested that administration of ADMSCs may normalize the metabolism in CLP rats.

### Multivariate Statistical Analysis

**Figure 2:**
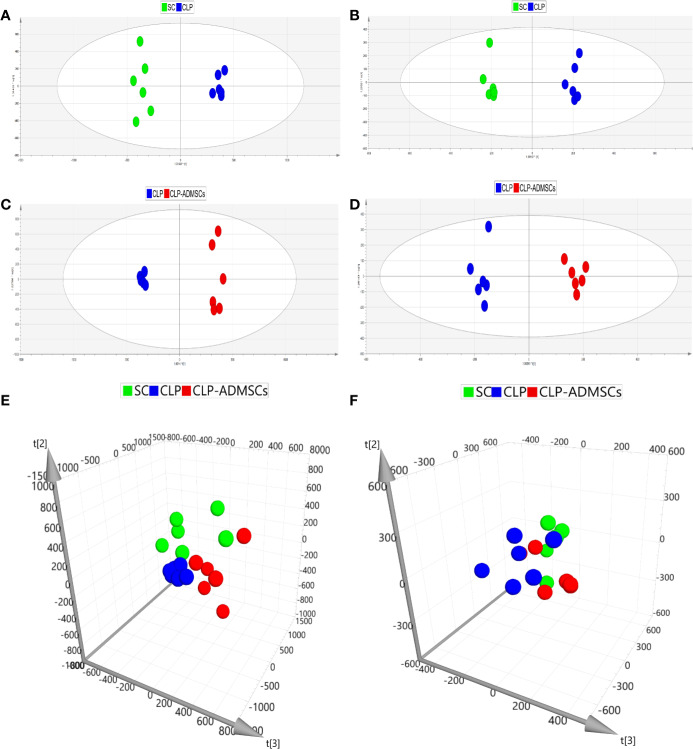
PCA and OPLS-DA scores of plasma samples (n = 6). **(A, C, E)** Positive ion mode. **(B, D, F)** Negative ion mode.

In order to make the analysis results more intuitive and visual, the unsupervised PCA method was applied. **Figures 2E, F** and **Supplementary Material Figures 3E, F** showed the three-dimensional score charts in plasma and lung tissues samples. The metabolite pattern changed significantly both in the positive and negative ion modes, and the CLP group was significantly different from the SC and CLP-ADMSCs groups. The CLP-ADMSCs group showed a significant callback trend both in plasma and lung tissues. Considering the overall change track of metabolites, it can be concluded that ADMSCs had a therapeutic effect on septic rats. In addition, the relative proximity of CLP-ADMSCs group and SC group indicated that ADMSCs had little effect on the metabolism of normal rats.

The OPLS-DA method was used to analyze the experimental data (**Figures 2A–D** and **Supplementary Material Figures 3A–D**). The scores of the CLP, SC, and CLP-ADMSCs groups were clearly different both in the plasma and lung samples, which showed that the metabolism profile changed significantly after ADMSCs treatment.

### Identification of Differential Metabolites

**Figure 3:**
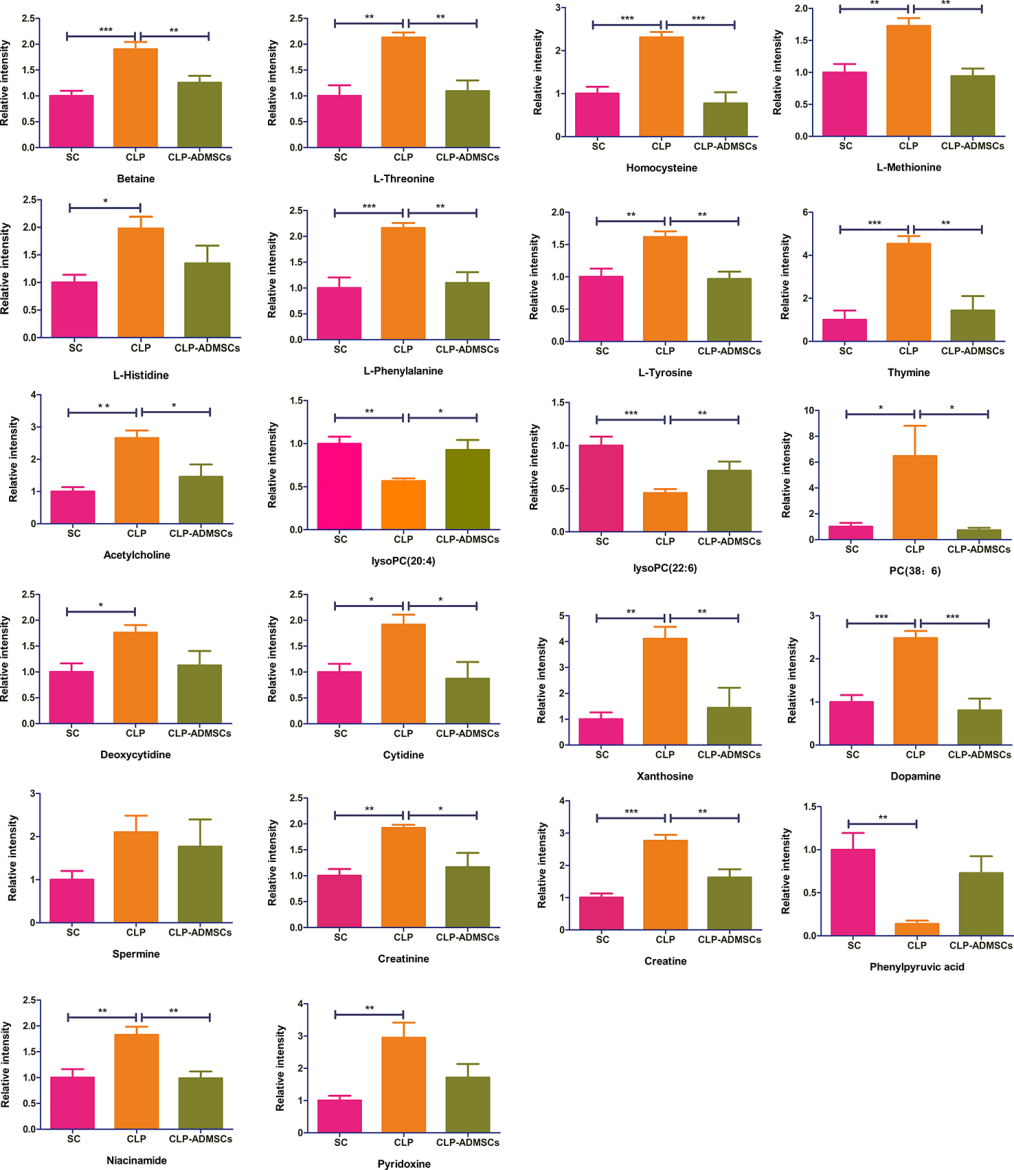
Relative intensities of major differential metabolites in the plasma (n = 6). *P < 0.05; **P < 0.01; ***P < 0.001.

**Figure 4:**
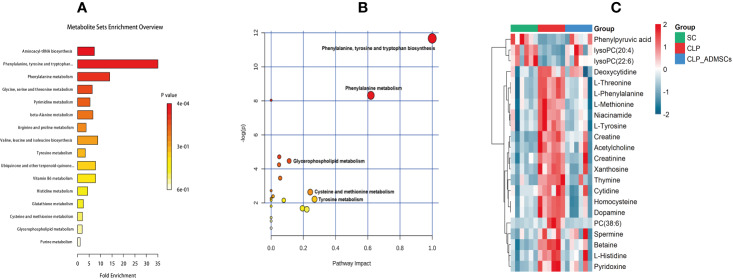
Diagram of differential metabolic pathways and enrichment of differential metabolites in lung tissues (n = 6). **(A)** Diagram of differential metabolic pathways (n = 6). **(B)** Enrichment of differential metabolites (n = 6). **(C)** Heat map of major differential metabolites in plasma (n = 6).

The VIP, FC, and *P* values were included in the volcano plot to find differential metabolites and coding scripts were available in Github website (https://github.com/cuiyuqing2018/cuiyuqing). *P* < 0.05 (–log_10_ (*P*-value) > 1.30), |FC| > 1.5 or < 2/3 (log_2_(FC) ≥ 0.585 or log_2_(FC) ≤ −0.585) and VIP > 1.0 were considered statistically significant differences, as shown in **Supplementary Material Figures 4A–D** and **Supplementary Material Figures 5A–D**). The results of searches in the HMDB, the KEGG, and the MetaboAnalyst databases showed 22 and 11 differential metabolites in the plasma and lung tissues samples, respectively [**Table 1** and **Supplementary Material** (**Supplementary Table 1**)]. Among the main differential metabolites in the CLP group, the levels of plasma creatinine, betaine, threonine, niacinamide, thymine, creatine, homocysteine, acetylcholine, methionine, dopamine, histidine, phenylalanine, pyridoxine, tyrosine, spermine, deoxycytidine, cytidine, xanthosine, and phosphatidylcholine [PC (38: 6)] were found to be increased, and those of lysophosphatidylcholine and phenylpyruvate significantly decreased, compared with SC group. However, ADMSCs treatment regulated 22 identified metabolites to levels similar to SC group (**Figure 3**). Moreover, altered metabolic pathways mainly included biosynthesis of phenylalanine, tyrosine, and tryptophan, phenylalanine metabolism, tyrosine metabolism, cysteine, and methionine metabolism, glycerol phospholipid metabolism (**Figures 4A, B**). Compared with the SC group, L-valine, creatine, acetylcholine, glutamic acid, xanthine, tryptophan, cytidine, lysophosphatidylcholine [LPC (18: 3), LPC (22: 4)], and chenodeoxycholic acid (CDCA) significantly increased in the CLP group of lung tissues samples. Further, phosphatidylcholine [PC (36: 4)] was decreased (**Supplementary Material Figure 6**). Altered metabolic pathways mainly included pathways for glycerol phospholipid metabolism, arginine, and proline metabolism, D-glutamine, and D-glutamic acid metabolism (**Supplementary Material Figure 7**).

**Table 1:** Major differential metabolites in septic rat plasma (n = 6).

NO	Differential metabolites	m/z	RT(min)	VIP	*p* value	FC(CLP/SC)	Involved pathways
1	Creatinine	114.0675	0.85	1.89	0.00007	1.92	10
2	Betaine	118.0869	0.79	6.23	0.0004	1.90	4
3	L-Threonine	120.0813	1.85	8.17	0.0005	2.13	2,4,7
4	Niacinamide	123.0455	1.06	1.37	0.0043	1.83	Nicotinate and nicotinamide metabolism
5	Thymine	127.0376	1.07	1.08	0.0001	4.53	4
6	Creatine	132.0774	0.89	10.05	0.00001	2.77	4, 10
7	Homocysteine	136.0497	0.88	2.23	0.00007	2.31	14
8	Acetylcholine	146.119	0.91	2.06	0.0001	2.66	15
9	l-Methionine	150.0595	1.03	2.93	0.002	1.73	2, 14
10	Dopamine	154.0603	0.88	2.94	0.00007	2.48	11
11	l-Histidine	156.0783	0.76	1.22	0.004	1.98	2, 6, 12
12	l-Phenylalanine	166.087	1.85	9.5	0.0005	2.16	1, 3
13	Pyridoxine	170.0945	0.76	1.45	0.003	2.95	9
14	l-Tyrosine	182.0821	1.03	3.57	0.003	1.62	1,2, 3, 8, 11
15	Spermine	203.1524	0.9	1.02	0.029	2.10	6, 10, 13
16	Deoxycytidine	228.1005	1.04	1.19	0.006	1.76	5
17	Cytidine	244.082	1.02	1.17	0.004	1.92	5
18	Xanthosine	285.1309	0.89	1.85	0.0002	4.11	17
19	lysoPC(20:4)	544.3428	5.76	18.25	0.0005	0.57	15
20	lysoPC(22:6)	568.3436	5.73	7.72	0.003	0.55	15
21	PC(38:6)	806.5717	8.77	3.51	0.043	6.47	15
22	Phenylpyruvic acid	162.9591	0.68	1.34	0.0015	0.14	1, 3

Compared with CLP group, potential biomarker information was screened in CLP-ADMSCs group. FC, fold change; VIP, contribution rate of different substances to orthogonal partial least squares discriminant analysis (OPLS-DA) model construction. RT, retention time. Involved pathways:1. Phenylalanine, tyrosine and tryptophan biosynthesis; 2. Aminoacyl-tRNA biosynthesis; 3. Phenylalanine metabolism; 4. Glycine, serine and threonine metabolism; 5. Pyrimidine metabolism; 6. beta-Alanine metabolism; 7. Valine, leucine and isoleucine biosynthesis; 8. Ubiquinone and other terpenoid-quinone biosynthesis; 9. Vitamin B6 metabolism; 10. Arginine and proline metabolism; 11. Tyrosine metabolism; 12. Histidine metabolism; 13. Glutathione metabolism; 14. Cysteine and methionine metabolism; 15. Glycerophospholipid metabolism; 16. Tryptophan metabolism; 17. Purine metabolism.

### Analysis of Metabolic Data

The heat map of coding scripts have been made available at Github website (https://github.com/cuiyuqing2018/cuiyuqing). The changing tendency of metabolites is shown in **Figure 4C** and **Supplementary Material Figure 8**). Obvious demarcation lines were found in the three groups. Multiple metabolites were clearly changed in the CLP group compared with SC group, but ADMSCs injection reversibly adjusted these parameters. And we found acetylcholine, spermine, phenylalanine, threonine of plasma, and PC (36:4) of lung tissues in CLP and CLP-ADMSCs groups were significantly associated with IL-6/TNF-α (**Table 2**).

**Table 2:** The correlation analysis between plasma IL-6/TNF-α and plasma/lung metabolites (n = 6).

Metabolites	IL-6 (CLP group)	TNF-α (CLP group)	IL-6 (CLP-ADMSCs group)	TNF-α (CLP-ADMSCs group)
Acetylcholine (plasma)	−0.83 (0.04)		−0.68 (0.14)	
Spermine (plasma)		0.98 (0.001)		0.69 (0.13)
Phenylalanine (plasma)		0.76 (0.08)	−0.75 (0.09)	
Threonine (plasma)		0.75 (0.08)	−0.74 (0.09)	
PC (36:4) (lung)	0.91 (0.01)	−0.77 (0.07)	−0.46 (0.36)	0.70 (0.12)

Each cell represents the correlation coefficient and P value. The correlation coefficient≥0.75 or P ≤ 0.2 were selected as differential metabolites associated with IL-6/TNF-α. PC, phosphatidylcholine.

## Discussion

In this study, PCA and OPLS-DA were used for the discriminant analysis, and the univariate statistical analysis was used to screen important differential metabolites in untreated or ADMSCs-treated septic rats. Metabolic analysis of plasma and lung tissues revealed that amino acid and glycerol phospholipid metabolism were altered in septic rats. We found four plasma metabolites and one lung metabolite associated with IL-6/TNF-α of plasma. These results suggested that ADMSCs may reduce anti-inflammatory factors, consequently reversing abnormal metabolic pathways in sepsis induced ALI. The findings might have vital effects on elucidating the mechanism of ADMSCs treatment in sepsis.

MSCs had been reported to attenuate ALI in animal models (Aslam et al., 2009; Su et al., 2019a). There is excessive inflammation in sepsis and ALI, and pulmonary infection is the lead site of sepsis (Hirano et al., 2018), with the lung being one of the first affected organs in sepsis patients (Abe et al., 2018). Reduced inflammatory factors could prevent pulmonary edema and neutrophil aggregation (Li et al., 2018; Yang et al., 2020). We evaluated H&E-stained lung sections and observed that ADMSCs therapy might reduce inflammatory cells infiltration and lung edema by decreasing IL-6/TNF-α in plasma. In addition, we also detected apoptotic cells in lung by terminal deoxynucleotidyl transferase dUTP nick end labeling (TUNEL) staining (Ding et al., 2019). Other studies reported IL-6, TNF-α, and H&E staining could assess the severity of ALI (Sun et al., 2011; Liu et al., 2018; Su et al., 2019b). Here, we demonstrated indicators that support ADMSCs-driven reduction of ALI induced by sepsis.

Various infection factors stimulate macrophages and neutrophils to produce IL-6, TNF-α, and other pro-inflammatory transmitters. Over expression of IL-6 and TNF-α could lead to excessive inflammatory response, which aggravate tissues damage and even systemic inflammatory response (Tanaka et al., 2016). Acetylcholine, spermine, phenylalanine, and threonine of plasma had significant association with IL-6/TNF-α in both CLP and CLP-ADMSCs groups (**Table 2**). These metabolites all had an increased tendency in CLP group, but showed decreased trend after ADMSCs injection. The vesicular acetylcholine transporter was up-regulated, accompanied by acetylcholine release in sepsis (Hoover et al., 2020). Activation of acetylcholine receptors could inhibit inflammatory response and ameliorate liver injury in sepsis (Wu et al., 2020). A previous study reported that ADMSCs expressed the M2 muscarinic receptor (Piovesana et al., 2018). Therefore, we inferred that ADMSCs might bind acetylcholine to reduce excessive inflammation. In addition, elevated phenylalanine is highly correlated with poor renal function and low albumin, which is also an independent predictor of death (Huang et al., 2019). It was reported that threonine and spermine expression are also increased in sepsis (Cui et al., 2018; Zahedi et al., 2019; Izquierdo-Garcia et al., 2019). Our findings suggested that ADMSCs might have therapeutic effect by reducing IL-6/TNF-α to lower phenylalanine, spermine, and threonine. Besides, phosphatidylcholine (36:4) (PC) of lung tissues had significant association with IL-6/TNF-α in both CLP and CLP-ADMSCs groups (**Table 2**). PC forms alveolar surfactant and cell membrane, and it is the most abundant phospholipid in human (Furse and de Kroon, 2015). Our results revealed that PC was down-regulated and LPC was up-regulated in lung tissues samples. The reduction of pulmonary surfactant had resulted in ALI of sepsis (Huang et al., 2005). Therefore, we speculated that PC and alveolar surfactants were degraded due to the release of IL-6/TNF-α in CLP group. ADMSCs treatment might restore alveolar surfactants and PC to normal, and these play a vital role on alleviating lung damage. The relationship between metabolites and inflammation markers suggested ADMSCs may reverse the altered metabolites by reducing inflammatory factors, thus playing a therapeutic role. Besides, these metabolites may be regulators of inflammatory response, and have the potential to serve as novel biomedicines for the treatment of sepsis.

**Figure 5:**
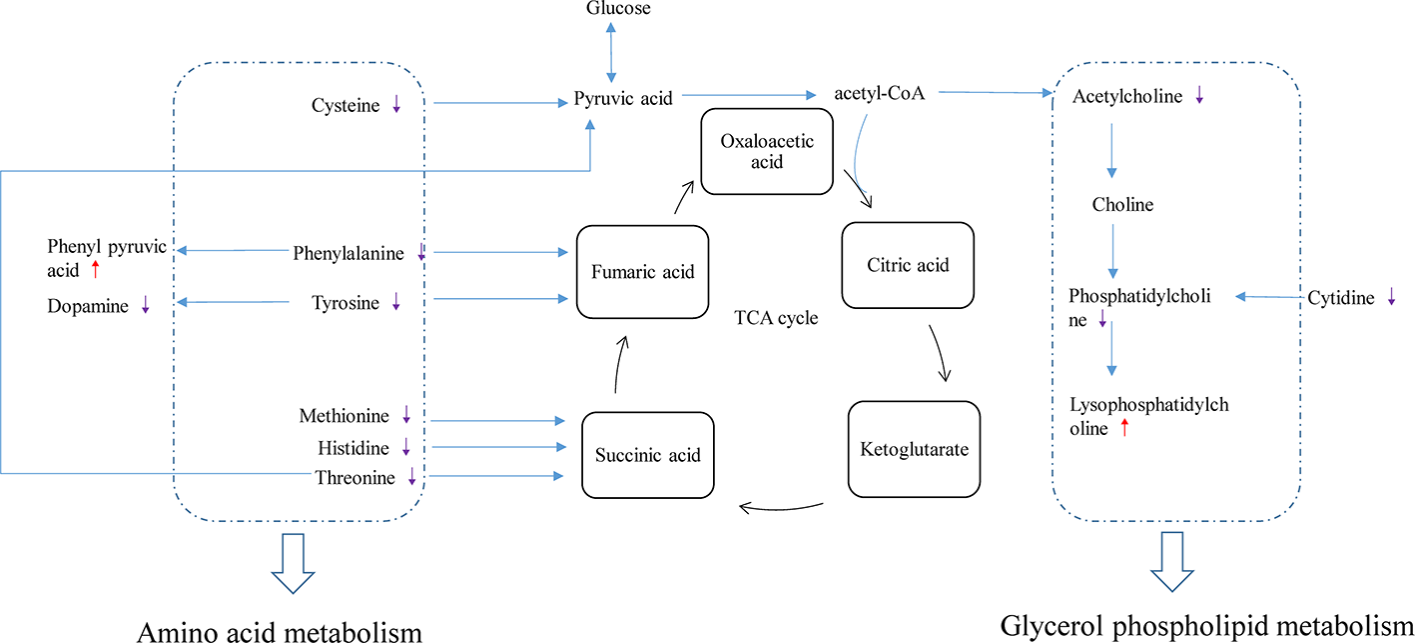
Disturbance of various pathways in plasma and effects of ADMSCs treatment (n = 6). The red arrow indicates increase in the metabolite concentration in the CLP-ADMSCs group relative to the CLP group, and the purple arrow indicates decrease in the metabolite concentration in the CLP-ADMSCs group relative to the CLP group.

Related physiological analysis had found sepsis experienced a highly catabolic status. A lot of protein decomposed into amino acids to supply energy, which seemed to be important for the poor prognosis (Van Wyngene and Vandewalle, 2018). Thus, the concentration of amino acids demonstrated a notable upward tendency in the CLP group. However, this state was reversed and the amino acid concentration decreased in the CLP-ADMSCs group. We speculated that the tricarboxylic acid cycle may return to normal and elevated amino acids were used preferentially to provide energy after ADMSCs treatment (**Figure 5** and **Supplementary Material Figure 9**). This inference has been proved in other metabolic diseases, such as obesity and type 2 diabetes (Shree et al., 2019; Yu et al., 2019).

However, this study also had several limitations. The small number of rats used to detect the metabolic changes in each group. The finding should be validated in a larger group of models. Our experiments involved small animals; future clinical translational studies in humans would be necessary to assess the benefits of ADMSCs in clinical practice.

## Conclusion

This study was a preliminary investigation of relevant metabolic changes in CLP and ADMSCs treatment groups. Based on metabolomics, ADMSCs restored disordered metabolic pathways, such as amino acid and glycerol phospholipid metabolism, to normal in septic rats. ADMSCs might have beneficial effect by reducing anti-inflammatory factors, reversing abnormal metabolic pathways in sepsis-induced ALI. This study provides new insights on ADMSCs-based strategy for sepsis treatment.

## Data Availability Statement

All datasets generated for this study are included in the article/**Supplementary Material**.

## Ethics Statement

The animal study was reviewed and approved by the Animal Care and Use Committee of the Zhengzhou University.

## Author Contributions

TS, YC, and SL conceived the study. YC, SL, XZ, XDi, and HL contributed to the animal experiments. ZZ and JZ contributed to the data processing and analysis. YC and SL contributed to the writing of the article. XDu, DW, and GZ resolved controversies. ZY, JY, and TS revised the article. All authors contributed to the article and approved the submitted version. All authors agree to be accountable for all aspects of the work in ensuring that questions related to the accuracy or integrity of any part of the work are appropriately investigated and resolved.

## Funding

This study was supported by the Leading Talents Fund in Science and Technology Innovation in Henan Province (Grant No.194200510017), Provincial Ministry Co-construction Project from Medical Scientific and Technological Research Program of Henan Province (Grant No. SBGJ2018020), the “51282” Project Leaders of Scientific and Technological Innovative Talents from Health and Family Planning Commission in Henan Province (2016-32), and the Science and Technology people-benefit project of Zheng Zhou (2019KJHM0001).

## Conflict of Interest

The authors declare that the research was conducted in the absence of any commercial or financial relationships that could be construed as a potential conflict of interest.
