# Supplementary material for: Metabolomic Analysis of the Effects of Adipose-Derived Mesenchymal Stem Cell Treatment on Rats With Sepsis-Induced Acute Lung Injury
Source: Front Pharmacol. 2020 Jun 17;11:902. doi: 10.3389/fphar.2020.00902 (PMC7311761; doi:10.3389/fphar.2020.00902)

Supplementary Material


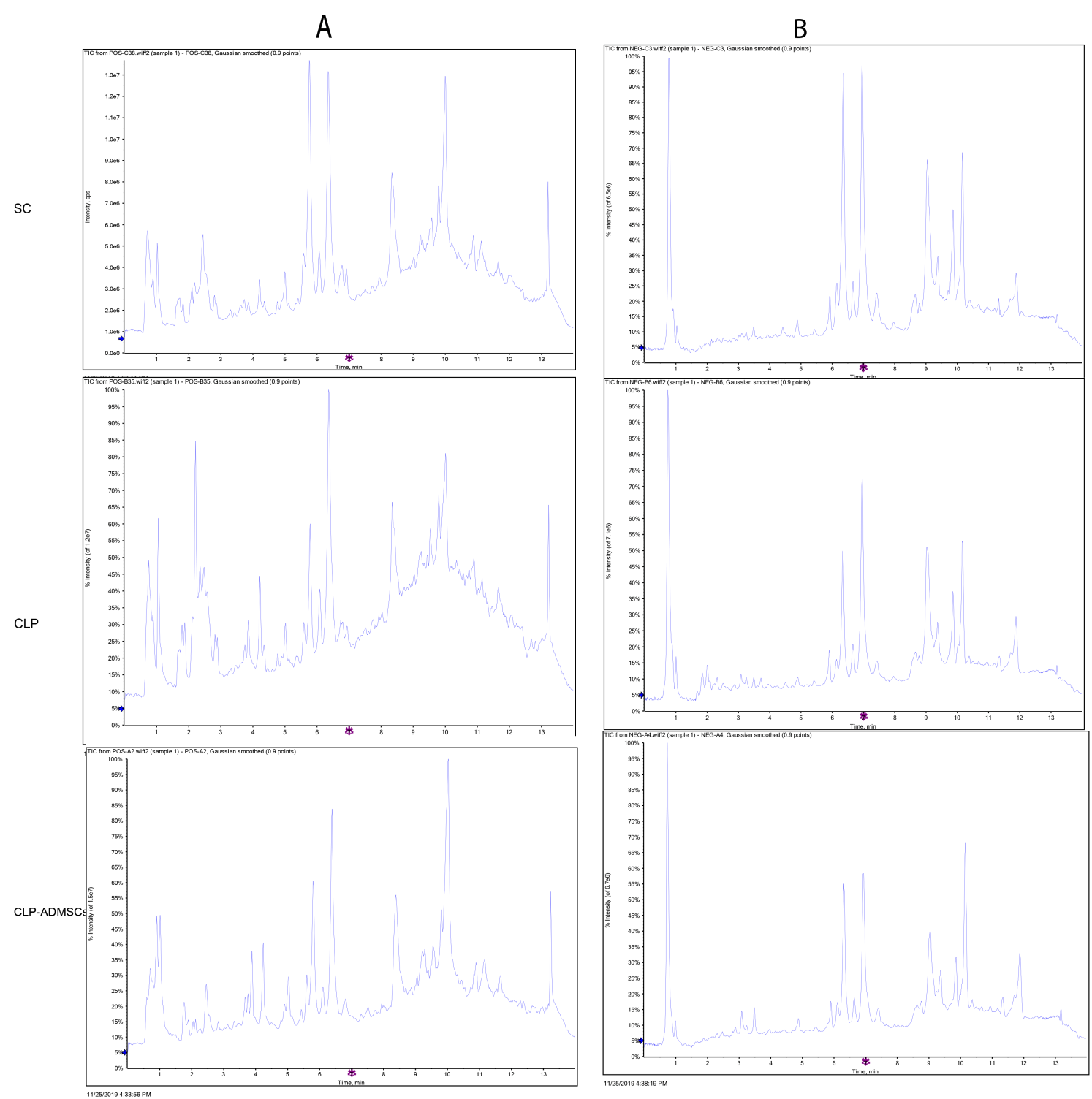
**Supplementary Figure 1.**Total ion chromatogram of plasma samples from SC, CLP and CLP-ADMSCs groups (n = 6). **(A)** Positive ion mode. **(B)** Negative ion mode.


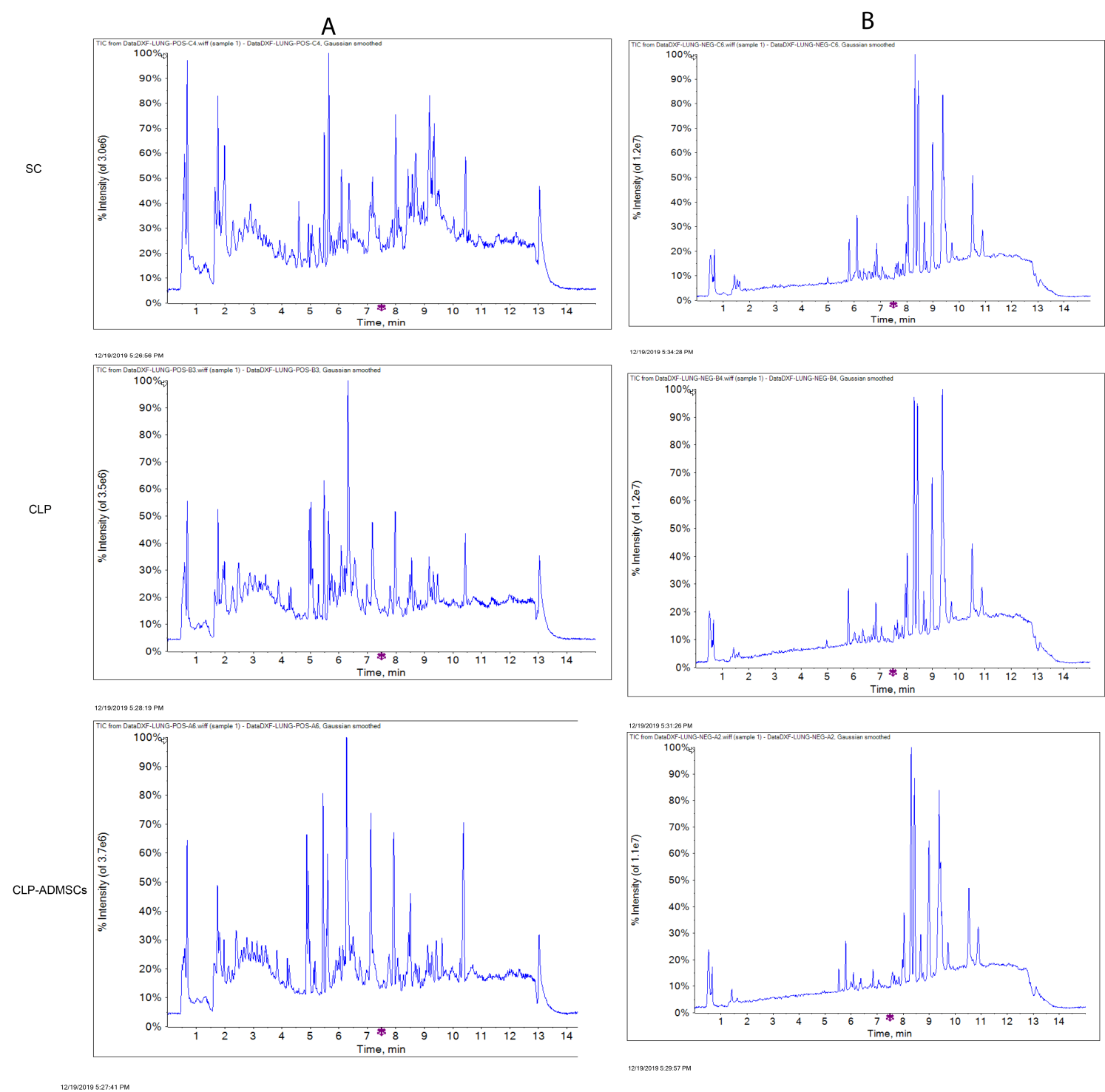
**Supplementary Figure 2.** Total ion chromatogram of lung samples from SC, CLP and CLP-ADMSCs groups (n = 6). **(A)** Positive ion mode. **(B)** Negative ion mode.


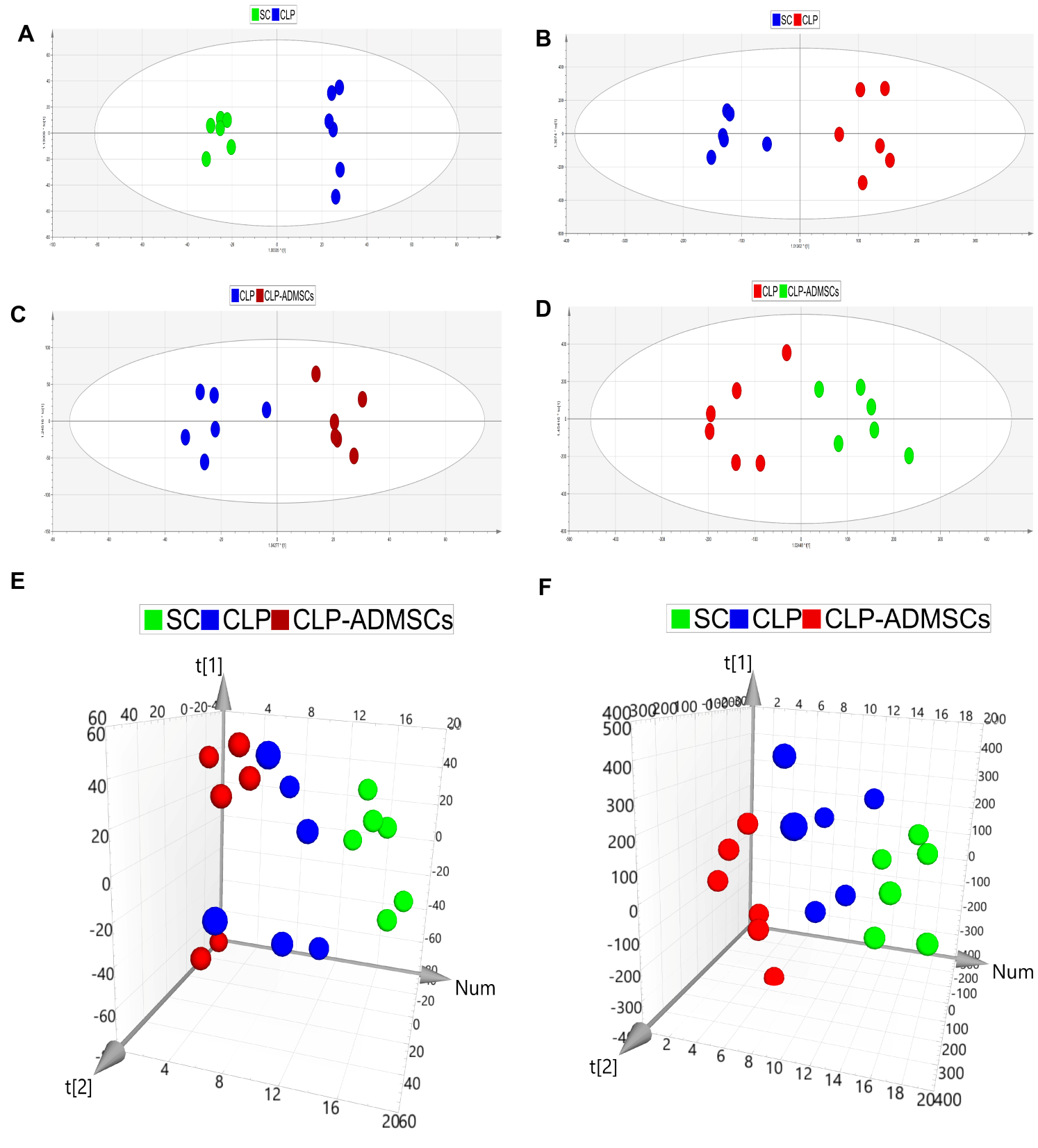
**Supplementary Figure 3.** PCA and OPLS-DA scores of lung tissues (n = 6). **(A)(C)(E)** Positive ion mode. **(B) (D) (F)** Negative ion mode.


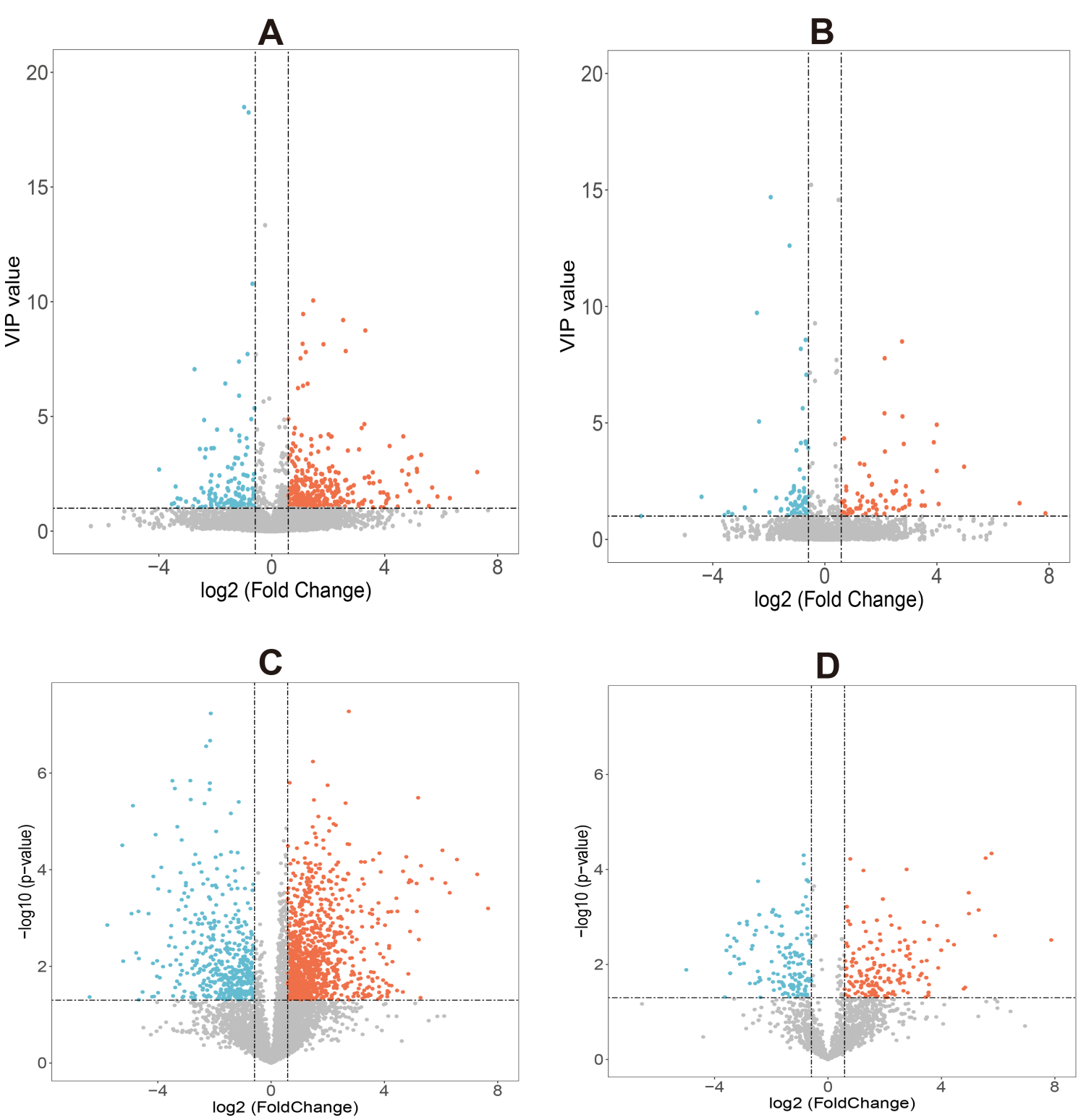
**Supplementary Figure 4.** Volcano plot of sepsis in plasma samples (n = 6). (**A) (C)** Positive ion mode. **(B) (D)** Negative ion mode.


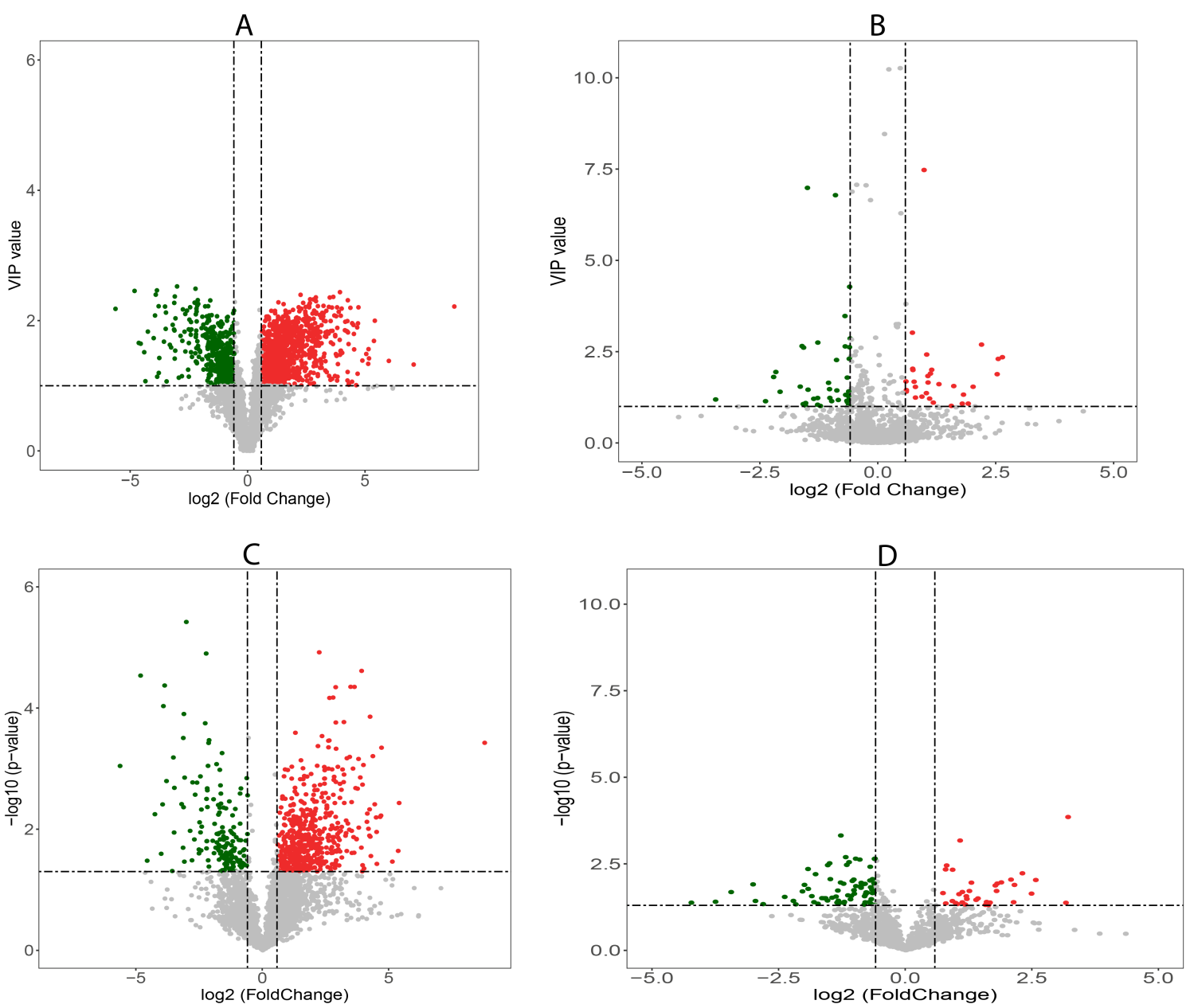
**Supplementary Figure 5.** Volcano plot of sepsis in the lung tissues (n = 6). **(A) (C)** Positive ion mode. **(B) (D)** Negative ion mode.


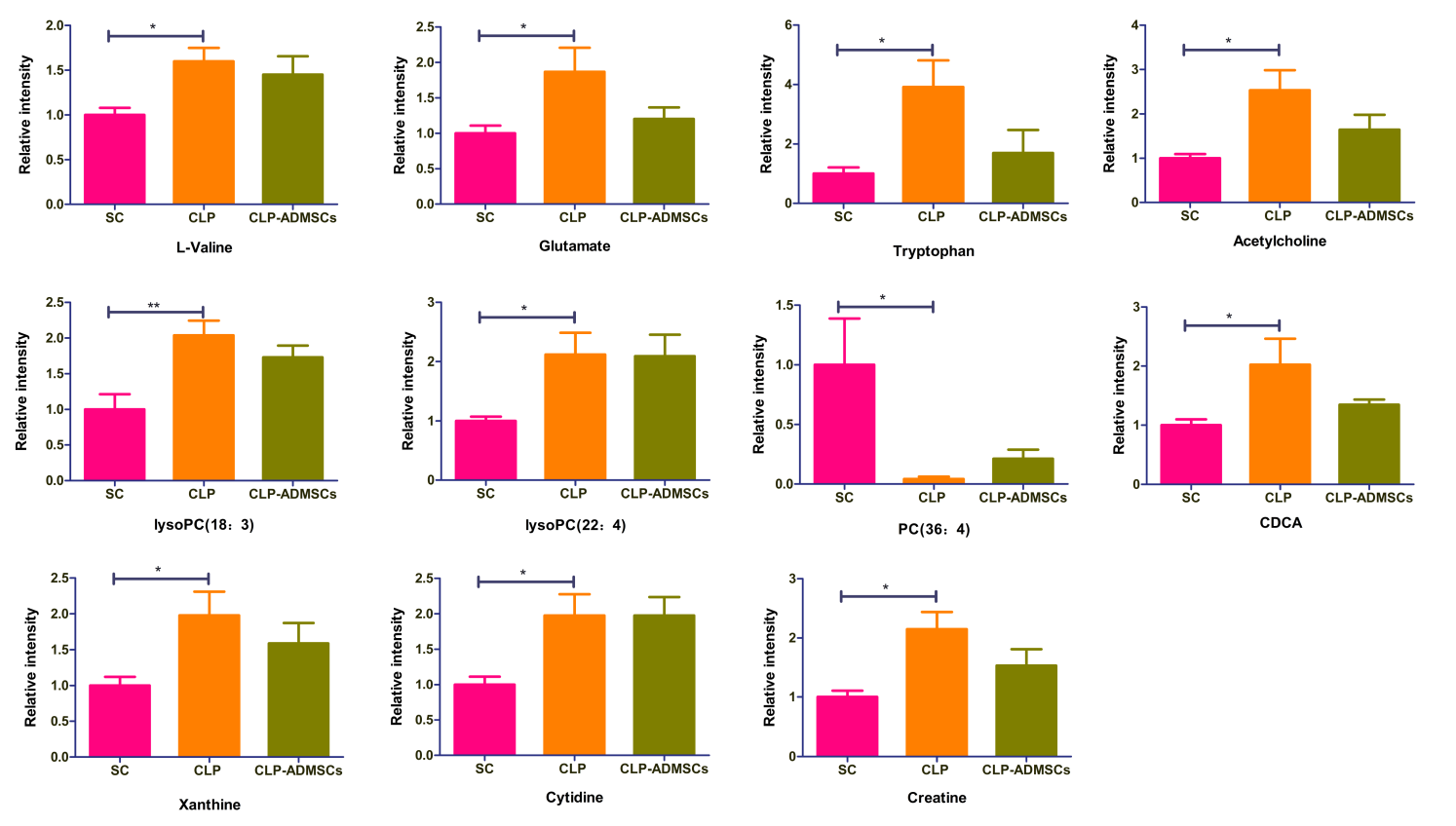
 **Supplementary Figure 6.** Relative intensities of major differential metabolites in the lung tissues (n = 6).


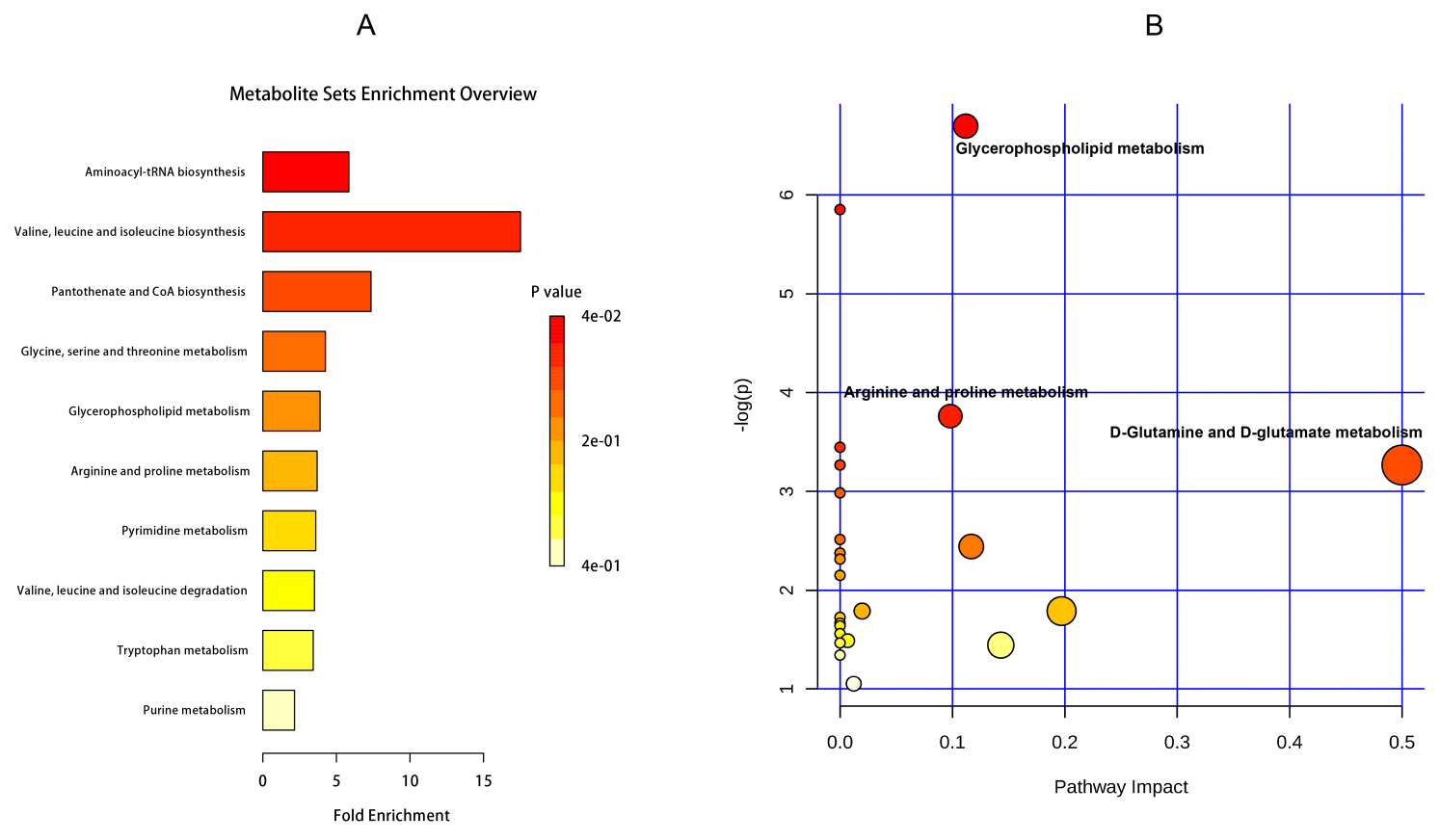
**Supplementary Figure 7.** Diagram of differential metabolic pathways and enrichment of differential metabolites in lung tissues (n = 6). **(A)** Diagram of differential metabolic pathways. **(B)** Enrichment of differential metabolites.


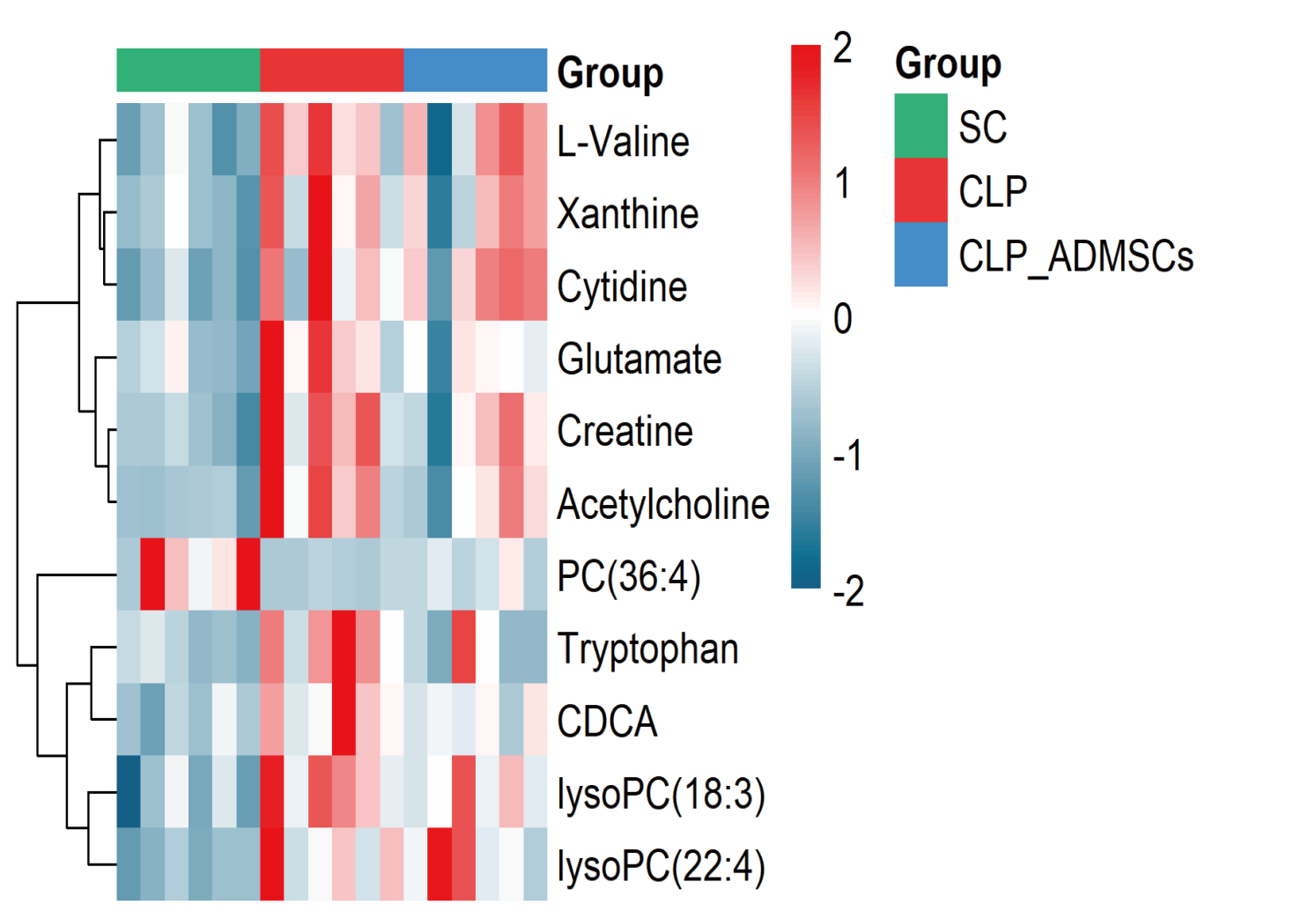
**Supplementary Figure 8.** Heat map of major differential metabolites in lung tissues (n = 6).

**
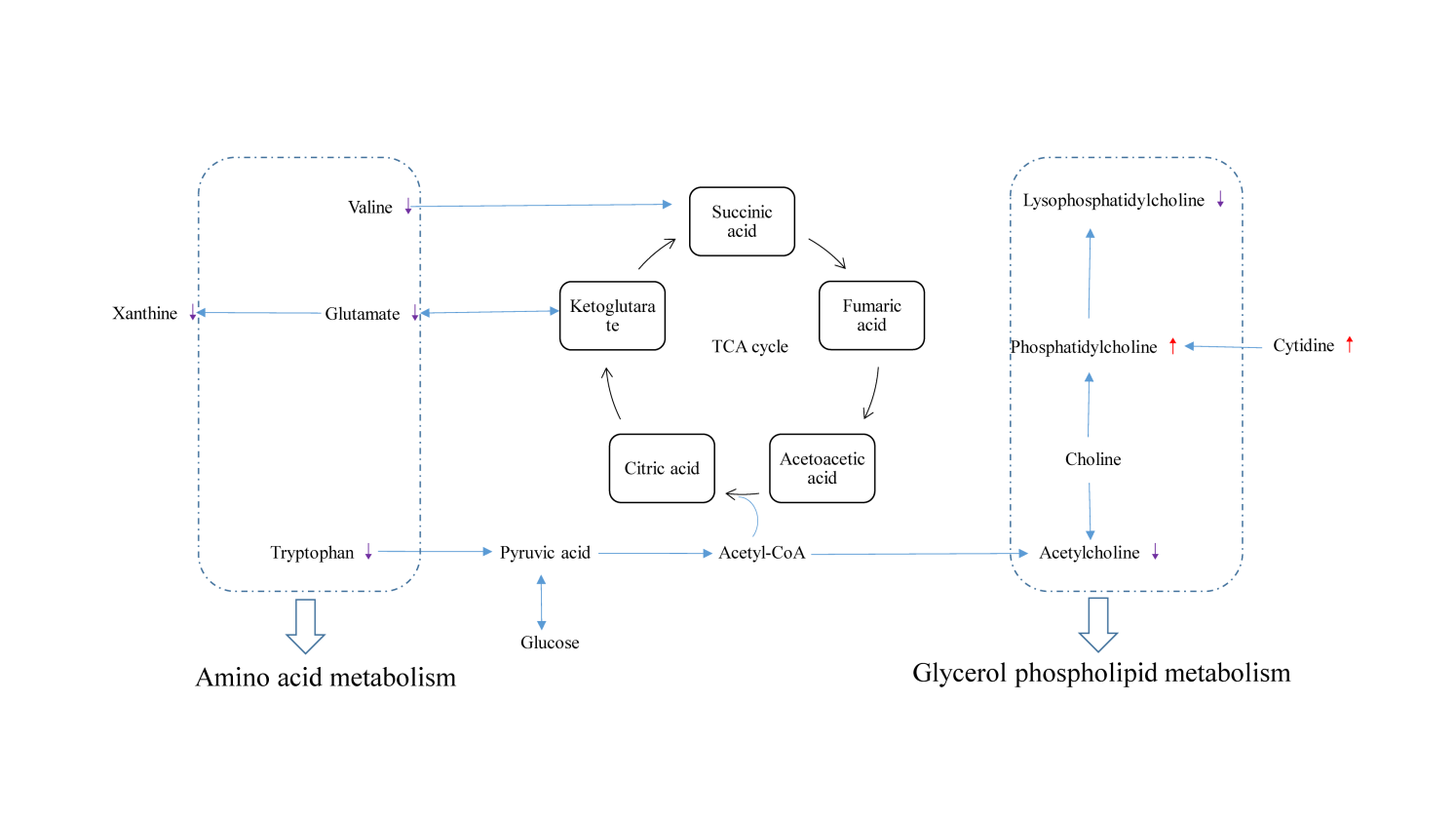
Supplementary Figure 9.** Disturbance of various pathways in the lung tissues and effects of ADMSCs treatment (n = 6). The red arrow indicates increase in the metabolite concentration in the CLP-ADMSCs group relative to the CLP group, and the purple arrow indicates decrease in the metabolite concentration in the CLP-ADMSCs group relative to the CLP group.

**Supplementary Table 1.** Major differential metabolites in the septic rat lung tissues (n=6).

| NO | Differential  metabolites | m/z | RT(min) | VIP | *p* value | FC（CLP/SC） | Involved pathways |
| --- | --- | --- | --- | --- | --- | --- | --- |
| 1 | L-Valine | 118.0863 | 0.71 | 1.90536 | 0.005371 | 1.60 | 2，7，21 |
| 2 | Creatine | 132.0763 | 0.58 | 1.93141 | 0.004068 | 2.15 | 3，18 |
| 3 | Acetylcholine | 146.1182 | 0.59 | 1.89105 | 0.007759 | 2.53 | 1 |
| 4 | Glutamate | 148.0613 | 0.55 | 1.5652 | 0.034771 | 1.87 | 2，3，5，6，10，11，12，14，15，16，17 |
| 5 | Xanthine | 153.0404 | 1.2 | 1.68563 | 0.019771 | 1.98 | 8 |
| 6 | Tryptophan | 205.0812 | 0.56 | 1.78511 | 0.010041 | 3.92 | 2，18 |
| 7 | Cytidine | 244.0937 | 0.85 | 1.7768 | 0.012189 | 1.98 | 20 |
| 8 | lysoPC(18:3) | 518.3222 | 5.67 | 1.91509 | 0.00569 | 2.04 | 1 |
| 9 | lysoPC(22:4) | 572.3709 | 6.64 | 1.77335 | 0.014041 | 2.12 | 1 |
| 10 | PC(36:4) | 782.5598 | 8.42 | 1.64315 | 0.033288 | 0.04 | 1,4，9，19 |
| 11 | CDCA | 391.2795 | 10.87 | 1.36011 | 0.046474 | 2.02 | 23 |

Note: Compared with CLP group, potential biomarker information was screened in CLP-ADMSCs group. FC, fold change; VIP, contribution rate of different substances to orthogonal partial least squares discriminant analysis (OPLS-DA) model construction; RT, retention time. Involved pathways:1. Glycerophospholipid metabolism; 2. Aminoacyl-tRNA biosynthesis; 3. Arginine and proline metabolism; 4. Linoleic acid metabolism; 5. Nitrogen metabolism; 6. D-Glutamine and D-glutamate metabolism; 7. Valine, leucine and isoleucine biosynthesis; 8. Purine metabolism; 9. alpha-Linolenic acid metabolism; 10. Arginine biosynthesis; 11. Butanoate metabolism; 12. Histidine metabolism; 13. Pantothenate and CoA biosynthesis; 14. Glutathione metabolism; 15. Alanine, aspartate and glutamate metabolism; 16. Porphyrin and chlorophyll metabolism; 17.Glyoxylate and dicarboxylate metabolism; 18. Glycine, serine and threonine metabolism; 19. Arachidonic acid metabolism; 20. Pyrimidine metabolism; 21. Valine, leucine and isoleucine degradation; 22. Tryptophan metabolism; 23. Primary bile acid biosynthesis.

**Appendix 1：**


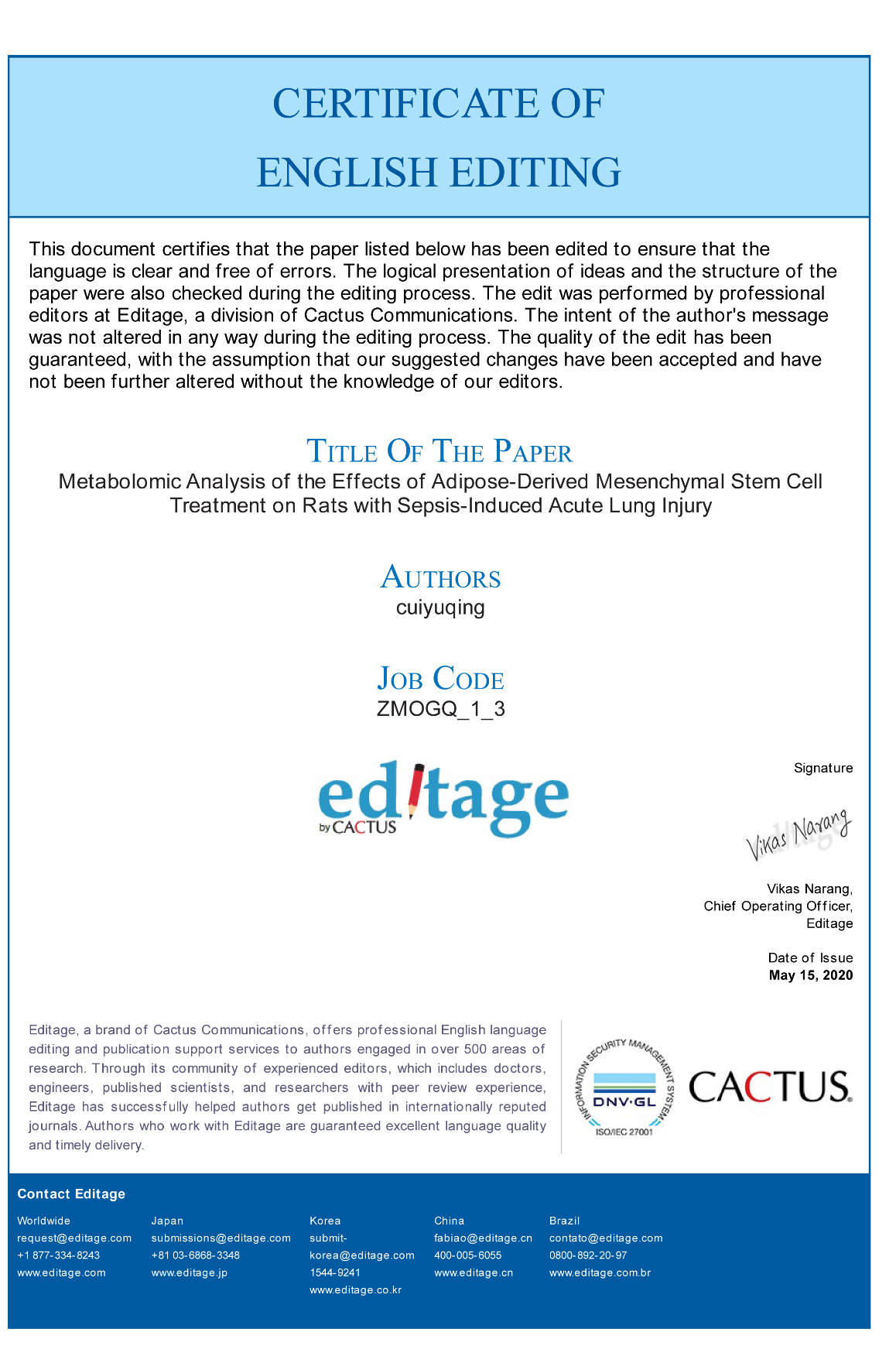

Supplement: Supplementary file 1 [file DataSheet_1.docx]
